# Association of fetuin-A with dyslipidemia and insulin resistance in type-II Diabetics of Pakistani population

**DOI:** 10.12669/pjms.36.2.1106

**Published:** 2020

**Authors:** Fasiha Fatima, Naveed Ahsan, Aliya Nasim, Faiza Alam

**Affiliations:** 1 Dr. Fasiha Fatima, MBBS, MPhil, Assistant Professor, Department of Biochemistry, Karachi Institute of Medical Sciences, CMH Malir Cantt, Karachi, Pakistan; 2 Dr. Naveed Ahsan, MBBS, MPhil, Assistant Professor, Department of Biochemistry, United Medical & Dental College, Karachi, Pakistan; 3 Dr. Aliya Nasim, MBBS, FCPS, Associate Professor, Department of Obstetrics & Gynecology, Liaquat College of Medicine & Dentistry, Darul Sehat Hospital Karachi, Pakistan.; 4 Dr. Faiza Alam, MBBS, MPhil, Senior Instructor, Department of Biological Sciences, Aga Khan University, Karachi, Pakistan

**Keywords:** Dyslipidemia, fetuin-A, Insulin resistance, Type-II diabetes mellitus mellitus

## Abstract

**Background & Objective:**

Fetuin-A, hepatokine is responsible for instigating insulin resistance by inhibiting tyrosine kinase receptors. Our objective was to investigate the relationship of fetuin-A with dyslipidemia and insulin resistance in type-II diabetics of Pakistani population.

**Methods:**

In this cross sectional study which was conducted at Basic Medical Sciences Institute, Jinnah Postgraduate Medical Centre, Karachi between October 2013 to March 2014, a total of 330 participants were selected and divided into two groups. Group-A (n = 165) normal healthy individual and Group-B (n = 165) Type-II diabetes mellitus mellitus with no comorbidities. Serum fetuin-A and insulin levels were determined by commercially prepared ELISA kits while fasting blood glucose (FBG) and lipid profile were performed by enzymatic kit method. Employing independent t-test, comparison of groups was done and correlation was achieved by using spearman correlation.

**Results:**

The results demonstrate a significant difference in mean values of fetuin-A, lipid profile, glucose, insulin and Homoeostasis Model Assessment of Insulin resistance (HOMA-IR) in type-II diabetics when compared to normal healthy individuals (p<0.01). A positive correlation was found between serum fetuin-A levels and FBG(r= 0.495, p< 0.001), insulin(r= 0.227, p< 0.001), HOMA-IR(r= 0.336, p<0.001, triglycerides(r= 0.197, p< 0.001) and LDL-cholesterol(r= 0.170, p= 0.002), while negative correlation with HDL-cholesterol(r= -0.251, p< 0.001).

**Conclusion:**

The study concludes that fetuin-A might be accountable for dyslipidemia and insulin resistance in type-II diabetes mellitus mellitus. So the high levels of Fetuin-A responsible for insulin resistance might alters endothelium and causes inflammation, vasoconstriction and thrombosis and ultimately atherosclerosis.

## INTRODUCTION

Type-II diabetes mellitus is complex metabolic disorder characterized by persistent hyperglycemia due to deficiency of insulin production or its resistance. Long term hyperglycemia is responsible for affecting the structure and functions of blood vasculature and tissues, which gives rise to complications like diabetic neuropathy, nephropathy, retinopathy, hypertension, hyperlipidemia, cerebrovascular diseases and atherosclerotic coronary heart disease.^1^ Serum lipid abnormalities (dyslipidemia) are commonly seen in diabetic populations irrespective of insulin deficiency or insulin resistance, might be contributing factor for macrovascular atherosclerosis disease and increased risk of cardiovascular disease.^2^

The majority of patients with type-II diabetes mellitus exhibit dyslipidemia, which is characterized by elevated triglycerides, low HDL-C and the predominance of small-dense LDL particles, however severe elevation in cholesterol is not observed frequently.^3^ Lipid abnormalities observed in type-II diabetes mellitus are not only quantitative, but also qualitative and kinetic in nature. Many factors may contribute to the changes in lipid metabolism in patients with type-II diabetes mellitus, including insulin resistance and/or relative insulin deficiency, adipocytokines (e.g. adiponectin), and hyperglycemia.^4^

Fetuin-A, also called Alpha 2-Heremans Schmid Glycoprotein (AHSG), is a multifunctional plasma factor secreted predominantly by hepatocytes.^5^ It is known to be a physiological inhibitor of insulin receptor tyrosine kinase^6^ and thus is associated with insulin resistance, metabolic syndrome (MetS) and an increased risk for type-II diabetes mellitus.^7^ Fetuin-A gene is positioned at the locus 27 on the (q) arm of chromosome 3, which is known to be a risk factor for type-II diabetes mellitus and cardio-metabolic disorders. Furthermore, genetic variants of fetuin-A were also related with type-II diabetes mellitus. The single nucleotide polymorphism (SNP) present at the 5’region of fetuin-A gene plays a role in regulation of lipid metabolism, conventionally facilitated by insulin. This highly advocates the chances of fetuin-A being involved in fat accumulation.^8^ Studies have shown a strong association between atherogenic lipid profile and fetuin- A. This association could be due to inhibitory effect of fetuin-A on adipose tissue, which leads to lipolysis and free fatty acid efflux.^9^,^10^

To best of our knowledge there is no data available for the association of fetuin-A with lipid profile and insulin resistance in local population. Thus the aim of our work was to study the relationship between fetuin-A with lipid profile and insulin resistance in patients with type-II diabetes mellitus mellitus in local population.

## METHODS

This cross-sectional case-control study was conducted at Basic Medical Sciences Institute, Jinnah Postgraduate Medical Centre, Karachi, between October 2013 to March 2014. The research protocol was approved by the institutional ethical committee of Basic Medical Sciences Institute Research (No. f.1-2/2013/bmsi-e.commt/004/Jpmc). The study protocol was in accordance with the principles expressed in the Declaration of Helsinki. All participants volunteered, and were elucidated about the research protocol. A complete verbal and written informed consent was obtained. The sample size was calculated by Open Epi software. Assuming a confidence level of 95%, power of 80%, least extreme odds ratio (OR) of 2 and a type-II diabetes mellitus mellitus prevalence of 11.77%,^11^ the sample size was calculated to be n=160, required to have a meaningful result of this study


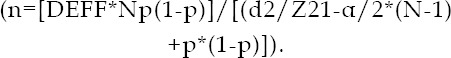


Our study was carried out on 165 known case of type-II diabetics between the ages of 35-60 years having less than 10 years of disease duration and on oral hypoglycemic drugs only (group B), from the Department of Medicine, JPMC, while 165 healthy individuals matched for age and sex were recruited from BMSI as controls (group A), with no comorbidities such as hypertension, infections, malignancies, chronic kidney disease etc. The diagnosis of type-II diabetes mellitus was made on basis of American diabetes association (ADA).^12^

Fasting serum glucose was estimated by GOD-PAP (Glucose Oxidase-Phenol-Aminophenazone) method (Merck, France). Fasting insulin was measured using an ELISA kit (DIA source Immuno Assay S.A., Belgium). Serum fetuin-A levels were measured with an enzyme immunoassay kit (DIA source Immunoassay S.A., Belgium Cat # KAPEPKT800), using ELISA plate reader equalizer ER 2005, (Eqiupar, Italy). Insulin resistance was calculated using the homeostasis model assessment of insulin resistance (HOMA-IR) index [fasting insulin (units per milliliter) x fasting glucose milligram/deciliter)/405].^13^ BMI was calculated by dividing weight by height meter squared (kg/m^2^).^14^ Cholesterol and HDL-C by CHOD-PAP (Cholesterol oxidase phenol Aminophenazone) method and Triglycerides by GPO (Glycerol-3-Phosphate Oxidase Phenol Aminophenazone) method, were measured by spectrophotometry (model AE-350, Erma Inc., Tokyo, Japan) using commercially available Merck, France kits. Low-density lipoprotein (LDL-C) was calculated by using Friedwald’s formula.^15^

### Statistical analysis

A statistical analysis of continuous variables was performed using SPSS (version 20; SPSS Inc., Chicago, IL, USA). Descriptive analysis of continuous variables i.e. biophysical (age, weight and BMI) and biochemical (Serum fasting glucose, insulin, cholesterol, triglycerides, HDL-cholesterol, LDL-cholesterol and fetuin-A) were calculated as mean ± standard deviation (SD). Statistical comparisons were computed using independent samples t-test for continuous/quantitative variables. Spearman’s correlation coefficient (r) was used to identify fetuin-A association with lipid profile and insulin resistance. P-value <0.05 was considered as significant.

## RESULTS

**Fig.1:**
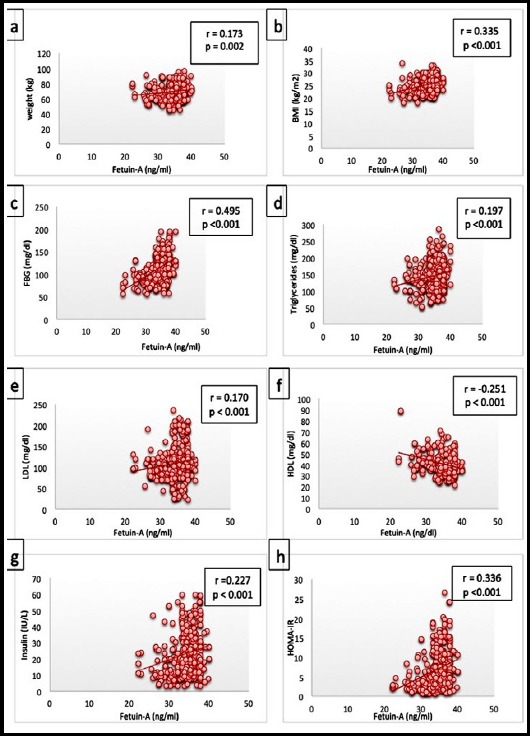
Correlation of Fetuin-A with **a)** weight, **b)** BMI, **c)** fasting blood glucose, **d)** triglcerides, **e)** LDL-cholesterol, **f)** HDL-Cholesterol, **g)** insulin and **h)** HOMA-IR.

A total of 320 age and gender matched subjects were selected. Group-A comprised of healthy control and Group-B comprised of known type-II diabetics. Table-I summarizes the clinical characteristics of the two groups showed significant difference in weight, BMI, FBG, cholesterol, triglyceride, LDL-cholesterol, HDL-cholesterol, insulin, HOMA-IR and fetuin-A. Age and cholesterol were not statistically significant. Fetuin-A correlated significantly positively with weight (r= 0.173, p= 0.002), BMI (r= 0.335, p< 0.001), FBG (r= 0.495, p< 0.001), triglycerides (r= 0.197, p< 0.001), LDL-cholesterol (r= 0.170, p= 0.002), HOMA-IR (r= 0.336, p<0.001) and insulin (r= 0.227, p< 0.001), while negatively correlates with HDL-cholesterol (r= -0.251, p< 0.001) (Fig.1 (a-h)).

**Table-I:** Biophysical and biochemical parameters of study subjects.

	Group-A (Healthy control) Mean ± SD (n= 165)	Group-B (Type-II Diabetics) Mean ± SD (n= 165)
Age (years)	52.02 ± 4.61	52.90 ± 4.78
Gender (male/female)	83/82	82/83
Weight (kg)	66.51 ± 9.66	72.08 ± 10.48 *
BMI (kg/m^2^)	23.58 ± 2.38	26.71 ± 2.73*
FBG (mg/dl)	90.75 ± 15.51	136.15 ± 17.60*
Cholesterol (mg/dl)	181.03 ± 35.74	186.89 ± 44.37
Triglyceride (mg/dl)	132.14 ± 33.71	156.56 ± 44.17*
LDL-cholesterol (mg/dl)	99.66 ± 33.71	119.94 ± 48.03*
HDL-cholesterol (mg/dl)	43.65 ± 8.99	38.47 ± 8.38*
Insulin (IU/L)	17.97 ± 10.98	28.43 ± 13.04*
HOMA-IR	4.13 ± 2.85	9.63 ± 4.90*
Fetuin-A (ng/dl)	32.37 ± 3.64	45.94 ± 1.93*

***Where:*** BMI (body mass index), FBG (Fasting blood glucose), LDL (Low density lipoprotein), HDL (High density lipoprotein) and HOMA-IR (Homeostasis model of insulin resistance) and

* reflects to p < 0.001.

## DISCUSSION

Type-II diabetes mellitus mellitus now serves as a great health hazard to the world. It continues to increase at an alarming rate, and is now 11.77% in Pakistan. The resistance of insulin is responsible for the majority of defects that lead to the development of type-II diabetes mellitus. Fetuin-A blocks the insulin from attaching to its receptors, creates a pathway and causes insulin resistance, and in this data it indicates that fetuin-A may play a role in pathophysiology of type-II diabetes mellitus. It has effect on the bodily defense mechanism involving deactivation of macrophages, rendering itself a diagnostic tool for metabolic syndrome.^16^

Our data shows that fetuin-A is found to be higher in type-II diabetics, when compared to healthy subjects. Other studies also suggested that fetuin-A might be responsible for development of type-II diabetes mellitus due to disturbance in regulation of insulin sensitivity, inflammation, non-alcoholic fatty liver disease and dyslipidemia.^17^-^20^ Another study prompts the role of fetuin-A in the metabolic changes occurring in pathogenesis of gestational diabetes.^21^ However, another study found no reference to fetuin-A level with insulin resistance, instead explaining their findings by the toxicity of glucose or protein modification such as the non-enzymatic glycation that may overcome the effect of fetuin-A on insulin resistance.^22^ Our study showed that BMI was highly significant in diabetics and was associated with serum fetuin-A levels, indicating that increase in levels may be held responsible for obesity, which is in line with other studies also.^19^,^24^ However, one study found high fetuin-A levels in subcutaneous adipose tissues of obese diabetics not in circulation suggesting that adipose tissues acts as sponge for storage of fetuin-A by temporary protection against excessive level in circulation.^23^

A major complication of type-II diabetes mellitus is dyslipidemia. Positive correlation to triglycerides, LDL-cholesterol has been found, as well as significant negative correlation to HDL-cholesterol with fetuin-A levels, which is consistent with findings of other researchers.^25^ Present study shows that fetuin-A level is associated with atherogenic lipid profile, without the history of cardiovascular diseases. According to this, it is postulated that fetuin-A might alter in lipid profile, which either directly or indirectly affects tyrosine kinase activity at insulin receptor. Nonetheless, direct correlation between serum cholesterol and fetuin-A concentrations could not be found. Yin et al., assess the effect of fetuin-A on vascular complication in new onset type-II diabetes mellitus mellitus and he found positive correlation with carotid intima media thickness, triglycerides, low density lipoprotein-cholesterol, HOMA-IR and fasting glucose.^26^ Another study clearly explained that role of fetuin-A in developing atherosclerosis by finding high levels of fetuin-A in atheromatous plaques of coronary arteries.^27^ We also found positive correlation of fetuin-A with insulin resistance, which is in line with another study.^28^ So the high levels of fetuin-A responsible for insulin resistance might alters endothelium and causes inflammation, vasoconstriction and thrombosis and ultimate atherosclerosis. This intimates that fetuin-A may provide an interesting link between dyslipidemia, insulin resistance and risk of cardiovascular disease in type-II diabetic patients.

### Limitation of the study

Our study is limited in terms of its small sample size and unable to study the effect of various oral hypoglycemic drugs on serum fetuin-A.

## CONCLUSION

Our results indicate that elevated fetuin-A levels correlating with insulin resistance and dyslipidemia might be responsible for instigating atherosclerosis by causing the atherogenic lipid profile. It raises the possibility that fetuin-A could be a potential therapeutic target in treatment of type-II diabetes mellitus mellitus and may be contributing factor in incidence of coronary artery disease.

### Authors’ Contribution:

**FF & FA** conceived, designed and did statistical analysis.

**NA & AN** did data collection and manuscript writing.

**FA** takes the responsibility and is accountable for all aspects of the work in ensuring that questions related to the accuracy or integrity of any part of the work are appropriately investigated and resolved. All authors were involved in editing the final manuscript.
